# Influence of *Suillus grevillea* on the Root Morphology, Growth and Rhizosphere Soil Properties of *Quercus variabilis* Blume Seedlings with Root Pruning

**DOI:** 10.3390/jof12010006

**Published:** 2025-12-21

**Authors:** Jinhua Sun, Shu Zhao, Liu Yang, Yazhen Liang, Xitian Yang, Lianfeng Shen, Erhui Guo, Qingxin Li, Yishuo Jia, Lin Zhang, Haoran Liu, Ruiling Sun

**Affiliations:** 1 Forestry College, Henan Agricultural University, Zhengzhou 450046, China; sunjinhua031234@henau.com.cn (J.S.);; 2 Postdoctoral Research Station of Landscape Architecture, Henan Agricultural University, Zhengzhou 450046, China; 3 Puyang County Branch of Puyang Ecological Environment Bureau, Puyang 457002, China

**Keywords:** root pruning, *Suillus grevillea*, root morphology, root physiology, soil properties

## Abstract

Root pruning affects the ability of roots of *Quercus variabilis* Blume to absorb water and nutrients. *Suillus grevillea* can form a mutualistic symbiosis with *Quercus variabilis* Blume. A pot experiment in three compartments with two inoculation treatments (inoculation with *Suillus grevillea* and noninoculation control) and four different root pruning treatments (0, 1/4, 1/3, and 1/2 of the main root length pruned) was conducted. The shoot dry weight, root dry weight, shoot and root N, P and K contents, root morphological and physiological parameters of *Quercus variabilis* Blume seedlings, and soil properties were measured. The results showed that root pruning affected root endogenous hormone levels, root morphology, shoot and root nutrient absorption, and biomass accumulation. Compared with those without inoculation, the shoot dry weight, root dry weights, shoot and root N, and P and K contents of inoculated plants were greater, regardless of the degree of root pruning. The root length, root projection area, root surface area, root average diameter, root density, root volume, and root tip number increased in response to *Suillus grevillea*. The root auxin (IAA), cytokinin (CTK), gibberellin (GA), zeatin riboside (ZR), and salicylic acid (SA) contents were greater in inoculated *Quercus variabilis* Blume seedlings than in noninoculated plants. Inoculation with *Suillus grevillea* improved the soil microenvironment around the seedlings. *Suillus grevillea* can compensate for the adverse effects of root pruning on nutrient absorption, root morphological and physiological growth and the soil properties of *Quercus variabilis* Blume seedlings.

## 1. Introduction

*Quercus variabilis* Blume belongs to the Quercus genus and the Fagaceae family and is a deciduous broad-leaved tree species [1]. It is a major afforestation tree species in the warm temperate zone of China because of its strong adaptability, rapid growth, and drought tolerance. These species play important ecological roles in the vegetation restoration of mountainous areas [2]. Quercus variabilis Blume exhibits drought and cold tolerance, adapts well to poor soils, and possesses ecological functions such as soil and water conservation, windbreak and sand fixation, climate regulation, water conservation, and providing habitats for flora and fauna [3]. Root pruning is a common method used in the afforestation process [4] and stimulates new root growth and lateral root regeneration [5,6]. However, previous studies have demonstrated that root pruning impedes root morphology development and plant growth [7]. Root pruning damages the root system of *Quercus variabilis* Blume seedlings during emergence, transplantation, and transportation. After root pruning, the survival rates of *Quercus variabilis* Blume seedlings decrease, growth and nutrient absorption are restricted, and photosynthesis decreases [3,8]. Therefore, root pruning is a stress factor for the growth of seedlings.

The effect of root pruning on plant growth is related to the intensity, method, time of pruning and plant species [9]. Light root pruning results in the formation of callus tissue in the damaged root system, stimulating lateral root growth, whereas severe root pruning reduces the ability of roots to absorb water and nutrients and impedes plant growth [10]. Moderate root pruning promotes the development of lateral roots in seedlings [4]. Therefore, the impact of different root pruning intensities on the physiological growth of plants is a topic of interest in the fields of restoration ecology and root ecology.

In plants, root pruning causes cell death, tissue loss, nutrient loss, nutrient limitation, susceptibility to pathogen invasion, and even plant death [11]. In plants, strategies to respond to injury by pruned roots, which include activating defenses against microbial infection and repairing root tissue damage, have been constructed [12]. New root morphology and structure occur in various plant species and involve the promotion of root formation at the wounding sites of tissues, which are relayed by signaling components (endogenous hormones) and promote rapid auxin (IAA) biosynthesis [13]. IAA governs core developmental processes in new root growth, from cell fate transitions to adventitious root patterning [14]. Cytokinin (CTK) signaling is necessary for limiting auxin signaling to the stem cell niche and, thus, for proper adventitious root development. In addition, gibberellic acid (GA) plays a positive role in regulating de novo root regeneration, as mutants for GA biosynthesis or signaling exhibit delayed adventitious rooting with a defect in vascular proliferation [15]. Abscisic acid (ABA) plays a role in inhibiting cell division during the formation of new roots, while promoting root hair growth and improving root absorption efficiency [16]. During the growth and development of plant roots, zeatin riboside (ZR) can stimulate the development of lateral branches, thereby promoting plant rooting. In addition, zeaxanthin can affect the formation and quantity of plant root hairs, increasing the ability of plants to absorb water and nutrients. Through these effects, zeaxanthin can increase plant productivity and tolerance to environmental stress. Salicylic acid (SA) serves as the primary defense response hormone, mediating host resistance against various biotrophs. SA is rapidly biosynthesized and systemically distributed in response to bacterial challenge. Mounting evidence suggests that SA also functions in abiotic stress responses to coordinate plant development and fitness under adverse environmental conditions [17]. SA not only confers resistance against pathogens in leaf explants but also plays a crucial role in the early stages of new root regeneration [18].

Ectomycorrhizal fungi (ECMFs) can establish symbiotic associations with host plant roots, where hyphae form a fungal mantle on the surface of feeder roots and extend into the intercellular spaces of the root cortex, forming a Hartig net [19]. The host plant provides carbohydrates for ectomycorrhizal fungi, and ECMFs provide abundant nutrients to plants through hyphae and improve plant growth [20]. The extended hyphae of ECMF can promote root absorption of soil moisture and nutrients [21,22], increase the photosynthetic rate of plants by increasing the accumulation of photosynthetic pigments, CO_2_ diffusion and electron transfer in leaf chloroplasts [23], and promote the mobilization of soil phosphorus and nitrogen to increase the tolerance of plants to drought, pathogens and toxic heavy metals [24,25,26]. The *Suillus luteus* belongs to the *Suillaceae* family and *Suillus* genus. This species is distributed throughout China, growing on the forest floor within mixed coniferous and broadleaf woodlands and coniferous forests. Fruiting bodies appear in autumn, winter, and early spring, occurring either singly or in clusters. ECMF can drive immune and defense mechanisms by regulating plant hormones such as jasmonic acid (JA) and SA, increasing the contents of polyamines, phenolic compounds, and osmolytes of *Quercus variabilis* Blume, and increasing plant growth [27].

Studies have confirmed that inoculation with the Suillus luteus can alter the contents of endogenous hormones such as indole-3-acetic acid (IAA), gibberellic acid (GA), zeatin riboside (ZR), and abscisic acid (ABA) in its host plant Pinus massoniana, resulting in an enhanced ability to regulate endogenous hormones under drought stress, thereby helping plants absorb nutrients and increasing their resistance to stress from the external environment [28,29]. Similarly, studies have shown that inoculation with *Pisolithus tinctorius* improves the physiological performance and field adaptability of *Quercus suber* seedlings by increasing leaf area, photosynthetic pigment contents (chlorophyll a, chlorophyll b, carotenoids), photosynthetic rate, stomatal conductance, water use efficiency (WUE), and foliar nitrogen (N) acquisition, ultimately enhancing nursery seedling growth and boosting survival rate and height after field transplantation [30]. Cultivation and transplantation in pots are common afforestation methods [31]. The disadvantage of studying ECMF via the potted cultivation method is that it treats the plant roots and ECMF hyphae as a whole, and the soil nutrient changes impacted by hyphae are ignored. However, a three-chamber separate culture system can separate hyphae, mycorrhizal fungi, and root systems and help us understand the mechanism by which ECMF affects root pruning seedlings [5].

The rhizosphere refers to the zone surrounding plant roots, acting as the primary site for interactions between plants and soil microorganisms. Roots secrete organic compounds, including carbohydrates, amino acids, and organic acids, thereby creating a nutrient-rich environment that attracts a wide variety of microbial communities [32]. Microorganisms in the rhizosphere facilitate nutrient uptake, hormone regulation, and disease resistance in plants. In comparison to non-rhizosphere soil, rhizosphere microorganisms exhibit increased sensitivity to nutrient cycling dynamics. Plants actively recruit specific microorganisms in the rhizosphere to support their growth and adaptation [33]. Considering the critical regulatory role of rhizosphere soil properties in plant growth and adaptation, it is essential to investigate how *Suillus grevillea* influences the growth of *Quercus variabilis* Blume seedlings by modulating the rhizosphere soil properties surrounding their roots.

However, studies on the effects of *Suillus grevillea* on the root physiology and morphology of *Quercus variabilis* Blume seedlings with root pruning are rare. Therefore, we hypothesize that (1) inoculation with *Suillus grevillea* can mitigate the adverse effects of root pruning on *Quercus variabilis* Blume seedlings and (2) *Suillus grevillea* enhances seedling growth by regulating root endogenous hormones, improving rhizosphere soil properties, and thereby facilitating nutrient uptake. To test these hypotheses, the aim of this study was to explore (1) the effects of *Suillus grevillea* on the root morphological traits and growth of *Quercus variabilis* Blume seedlings with different degrees of root pruning and (2) how *Suillus grevillea* affects the growth of *Quercus variabilis* Blume seedlings with root pruning by regulating the rhizosphere soil properties of the seedlings.

## 2. Materials and Methods

### 2.1. Experimental Materials

*Suillus grevillea* was purchased from the China Forestry Microbial Strain Collection Center in May 2024 and was expanded and preserved in the microbiology laboratory of Henan Agricultural University. The microbial agent tested is an artificially propagated biological preparation, with the *Suillus grevillea* strain serving as its core active component. The preparation process involves subjecting PDA medium (formula: 200 g/L potato, 20 g/L glucose, 20 g/L agar, 5 g/L lactose) to autoclaving at 121 °C for 30 min, then cooling and pouring the mixture into agar plates. The activated *Suillus grevillea* strain was inoculated onto the agar plates and incubated in a constant-temperature incubator at 25 ± 1 °C. When the mycelium had covered the plates, it was transferred to potted substrates. Simultaneously, sterile PDA medium without mycelium was prepared as the noninoculated control microbial agent. The soil used in this study is commercially purchased and consists of nutrient-poor sandy material. The barren sandy soil was passed through a 2 mm sieve, sterilized at 120 °C for 2 h, dried, and set aside for later use. The basic physiochemical characteristics of the soil were as follows: the soil pH was 7.68, the soil conductivity was 0.13 mS/cm, the soil maximum water holding capacity was 20%, the soil available phosphorus content was 4.25 mg/kg, the soil available potassium content was 49.14 mg/kg, the soil organic matter content was 10.64 g/kg, and the soil alkaline nitrogen content was 0.43 g/kg. Cork oak seeds were collected from artificial forests of *Quercus variabilis* Blume in the Funiu Mountains.

### 2.2. Experimental Design

*Suillus grevillea* was purchased from the China Forestry Microbial Strain Collection Center in May 2024 and was expanded and preserved in the microbiology laboratory of Henan Agricultural University. The root pruning treatments included 0, 1/4, 1/3, and 1/2 of the main root length cut off, with 5 repetitions for each treatment, 1 (inoculation treatment) × 4 (root pruning treatments) × 5 (replicates) = 20 pots. The seeds of *Quercus variabilis* Blume were disinfected with 10% H_2_O_2_ on the surface, cleaned with deionized water, and placed in an incubator at 25 °C for germination and future use. A pot experiment was conducted at the Ecological Restoration Laboratory of Henan Agricultural University on 5 June 2024. The sterilized soil was placed in the seedling pots (length × width × height = 10 cm × 10 cm × 15 cm), and 2 seeds were planted in each pot. When three leaves grew, one vigorous seedling was retained.

**Figure 1:**
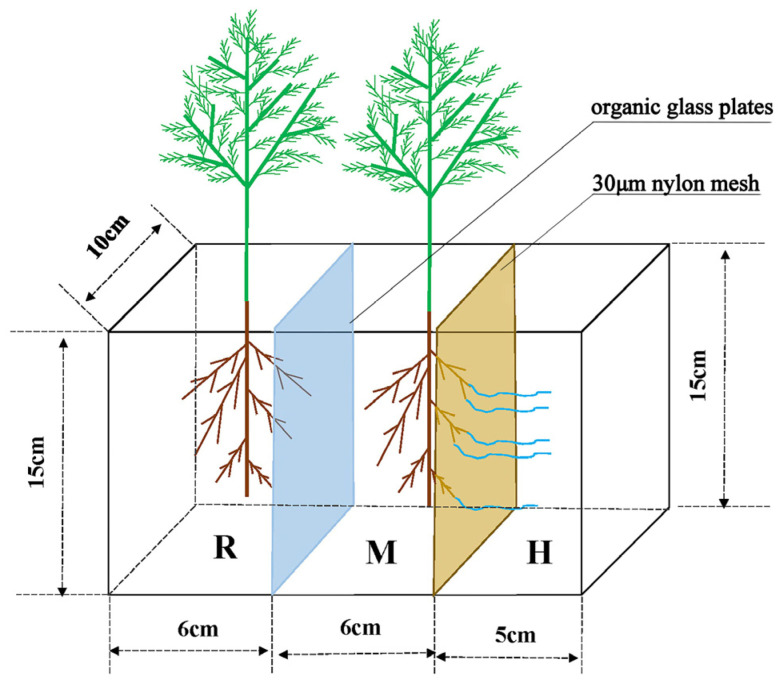
Diagram of three-chamber partition system apparatus. R–M, organic glass plate: both root and mycorrhizal cannot pass; M–H, 30 μm nylon mesh: mycelia can pass, mycorrhizal cannot pass.

After 20 days, the roots of *Quercus variabilis* Blume seedlings were approximately 10 cm long. Root lengths of 0, 1/4, 1/3, and 1/2 were cut off via blades disinfected with 75% alcohol, after which the seedlings were moved into a three-compartment separation system. The device material was organic glass, with dimensions of length × width × height = 17 cm × 10 cm × 15 cm. The left panel in Figure 1 shows the root compartment (R), the middle panel shows the mycorrhizal compartment (M), and the right panel shows the hyphal compartment (H). The R and M compartments were separated by organic glass plates, whereas the M and H compartments were separated by a 30 μm nylon mesh; the roots cannot pass through, but the hyphae can (as shown in Figure 1).

The three-compartment separation system was filled with sterilized sand. The inoculation and noninoculation treatments involved mixing 10% of the microbial agent and 10% of the sterile microbial agent, respectively, with the soil. The inoculation treatment was completed in the M compartment, whereas the noninoculated treatment was completed in the R compartment. Pot experiments were conducted in the greenhouse of the Ecological Restoration Laboratory at Henan Agricultural University under controlled conditions (25–35 °C, 12–14 h photoperiod), with consistent management of watering, light, ventilation, and temperature throughout the cultivation period. The first watering amount was the maximum water holding capacity of the soil, and the soil moisture content was maintained between 60% and 70% during the growth of *Quercus variabilis* Blume seedlings. Simultaneously, nutrient solutions prepared with NH_4_NO_3_, KH_2_PO_4_, and KNO_3_ were applied to achieve soil N, P, and K contents of 100, 30, and 150 mg/kg, respectively, in each compartment. After 60 days, the shoots and roots of the seedlings in the R and M compartments were collected.

### 2.3. Indicator Determination Method

The roots were subsequently washed with deionized water, the surface moisture on the roots was wiped dry with absorbent paper, and the root morphological parameters were measured. Some fresh roots were selected to measure mycorrhizal colonization and endogenous hormones, and the remaining roots and shoots were selected for biomass measurement. The dry shoot and root of the seedlings were ground and sieved through a 0.149 mm sieve for mineral element determination. The soil in the R, M, and H compartments was air-dried and sieved through a 2 mm sieve for physiochemical analysis.

#### 2.3.1. Biomass

The dry weights of the shoot and root of the seedlings were determined via the oven-drying method. The shoot and root were shaken in an oven at 105 °C for 30 min and dried to a constant weight at 75 °C for 72 h.

#### 2.3.2. Mycorrhizal Colonization

A microscope was used to examine the mycorrhizal colonization rate. Ten pieces of lateral roots were randomly sampled from the entire root system of each seedling and then completely washed with distilled water and stored in ethanol. The roots were immersed in Calcofluor white solution (0.2%, *w*/*v*) for a few seconds and then washed with distilled water for 30 s and placed on a glass slide. The tips of the roots stained with Calcofluor white were examined under a microscope at ×200 magnification [34]. The mycorrhizal colonization percentage (IMC) was calculated via Equation (1).

(1) IMC = [∑(mi × ni)/R] × 100

where mi is the relative value of mantle development in each root tip, ni is the number of root tips observed, and R is the total number of root tips observed in each root system.

#### 2.3.3. Root Morphology

The root system was rinsed with tap water and deionized water, wiped clean, and scanned via a root scanner (STD4800; EPSON, Suwa, Japan). The root morphology parameters, including the root length, root projection area, root surface area, average root diameter, root density, and root volume, were analyzed via WinRHIZO Pro 2007 software (Regent Instruments, Canada) [35].

#### 2.3.4. Root Endogenous Hormone Content

Fresh roots stored at −20 °C were accurately weighed into 0.5 g of 2 mL of PBS buffer (phosphate buffer, pH 7.5), and a small amount of quartz sand was added; the roots were ground in an ice bath, and the mortar was rinsed with 2 mL of PBS buffer. The grinding solution was transferred to a 10 mL centrifuge tube, extracted at 4 °C for 4 h in a refrigerator, and centrifuged at 4 °C and 10,000 r/min for 15 min, after which 2 mL of the supernatant was removed to measure the root IAA, GA, ABA, CTK, ZR and SA contents by an enzyme-linked immunosorbent assay (ELISA) kit (Shanghai, Enzyme Linked Biotechnology Company, China) [36].

#### 2.3.5. Nutrient Contents

The shoot and root nitrogen contents were measured via the wet chemical digestion/oxidation method of Kjeldahl. After the shoot and root of the seedlings were ground and sieved, 0.2 g was accurately weighed. The samples were boiled with H_2_SO_4_-H_2_O_2_, and the shoot and root phosphorus and potassium contents of the seedlings were determined via ICP‒AES (inductively coupled plasma emission spectrometer) [37].

#### 2.3.6. Soil Physiochemical Properties

The soil pH and soil conductivity were measured via a pH meter (water-to-soil mass ratio of 2.5:1) and a conductivity meter (water-to-soil mass ratio of 5:1), respectively. The soil alkaline hydrolysis nitrogen content was measured via the alkaline hydrolysis diffusion method, the NaHCO_3_ extraction molybdenum antimony colorimetric method was used to determine the soil available phosphorus content, and the flame photometry method was used to determine the soil available potassium content. The soil’s total carbon and nitrogen contents were determined via a carbon and nitrogen element analyzer [38].

### 2.4. PLS-SEM Modeling

Partial least squares structural equation modeling (PLS-SEM) is a variance-based multivariate statistical method that combines principal component analysis, canonical correlation analysis, and least squares to handle complex causal relationships and latent variables, particularly suitable for research scenarios involving prediction-oriented, small sample, or formative indicators. A complete PLS-SEM model consists of two parts, namely the external model (measurement model) and the internal model (structural model): the measurement model is used to express the relationship between observed variables and latent variables, and the structural model is used to express the relationship between exogenous latent variables and endogenous latent variables. Unlike covariance-based structural equation models (CB-SEM), partial least squares structural equation modeling (PLS-SEM) has been widely applied in social science research and has only recently been introduced into ecology and socioecology studies [39]. Given its ability to effectively address multicollinearity in complex systems and quantify the effects of multiple factors on target variables, PLS-SEM offers substantial analytical advantages for examining intricate causal relationships.

In this study, the latent variables are categorized into four groups: root physiological properties, soil physicochemical properties, root nutrition, and root morphological characteristics. The analysis aims to investigate the mechanisms through which root physiological properties and soil physicochemical properties influence biomass, either directly or indirectly, by regulating root nutrition and root morphology. To mitigate the potential adverse effects of excessive data variability on the final results, all data were standardized. Multiple indicators were employed to assess model quality: (1) Cronbach’s alpha ranges from 0 (completely unreliable) to 1 (completely reliable); (2) composite reliability should exceed 0.70; (3) the average variance extracted (AVE) should exceed 0.50; (4) factor loadings should exceed 0.50. If factor loadings or AVE values are low, indicating potential inconsistency among latent variables, the model can be improved by removing non-significant indicator variables. The goodness of fit (GoF) was used to evaluate the overall quality of the model [40].

### 2.5. Statistical Methods

The differences in the root morphological parameters, endogenous hormone contents, shoot and root biomass and nutrient contents, and soil properties among the different treatments were analyzed via two-way analysis of variance via SPSS 25.0 software (IBM Inc, USA). Duncan’s test for multiple comparisons was performed (significance level *p* = 0.05). The figures were drawn with Origin 9.1 software (Electronic Arts Inc, USA). The correlations among indicators, including aboveground biomass, root biomass, nutrient elements, endogenous hormones, and root morphology, were assessed by Pearson’s correlation coefficient.

## 3. Results

### 3.1. Mycorrhizal Colonization and Mycelial Density

**Figure 2:**
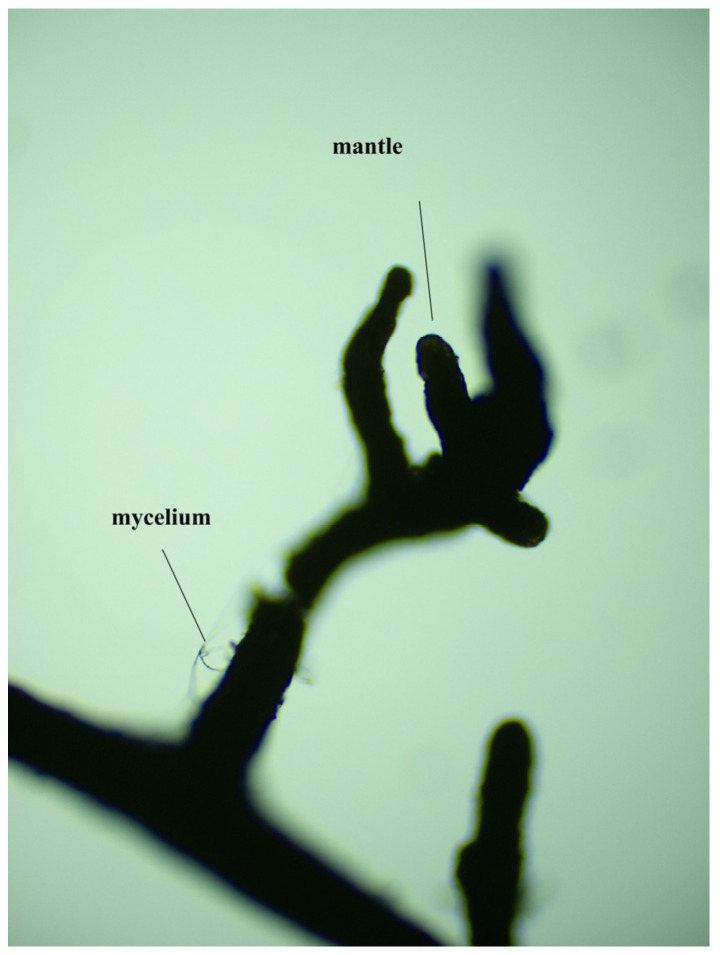
*Suillus grevillea* infection map.

As shown in Table 1, the mycorrhizal colonization and mycelial density of inoculated *Quercus variabilis* Blume seedlings decreased with increasing degree of root pruning. Compared with that of the seedlings without root pruning, the mycorrhizal colonization of the seedlings with 1/4, 1/3 and 1/2 root pruning significantly decreased (*p* < 0.05). The mycelial density of the seedlings with 1/4, 1/3 and 1/2 root pruning significantly decreased by 47.6%, 56.8% and 74.8%, respectively (*p* < 0.05). These findings indicate that root pruning disrupts mycorrhizal colonization and the extension of mycelia. Figure 2 shows that the *Suillus grevillea* infected the roots.

### 3.2. Biomass of Seedlings

**Figure 3:**
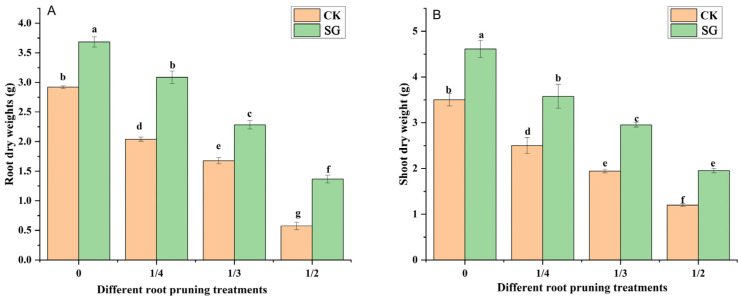
Effects of *Suillus grevillea* on the shoot and root dry weights of seedlings subjected to different root pruning treatments. (**A**) Effects of *Suillus grevillea* on the root dry weight of seedlings subjected to different root pruning treatments; (**B**) Effects of *Suillus grevillea* on the shoot dry weight of seedlings subjected to different root pruning treatments; CK, without inoculation; SG, inoculation with *Suillus grevillea*; the values 0, 1/4, 1/3 and 1/2 represent the removal of zero, one-fourth, one-third, and one-half, respectively, of the seedling taproot length; different lowercase letters in the figure indicate that mean values are significantly different (*p* < 0.05) according to the LSD test, while the same letter denotes no significant difference between treatments; error bars represent the standard error (SE) of the mean (n = 5).

The shoot dry weight (SDW) and root dry weight (RDW) reflect the biomass accumulation of the seedlings. As the degree of root pruning increased, the shoot and root dry weights of *Quercus variabilis* Blume seedlings gradually decreased (Figure 3A,B). Under 0, 1/4, 1/3 and 1/2 root pruning, the shoot and root dry weights of oak seedlings inoculated with *Suillus grevillea* were greater than those without *Suillus grevillea* inoculation. Compared with that of inoculated plants, the SDW of inoculated plants increased, and the RDW of inoculated plants significantly increased under the 0, 1/4, 1/3, and 1/2 root pruning treatments. Thus, inoculation with *Suillus grevillea* can alleviate the adverse effects of root pruning on seedling growth.

### 3.3. Mineral Nutrition of the Seedlings

**Figure 4:**
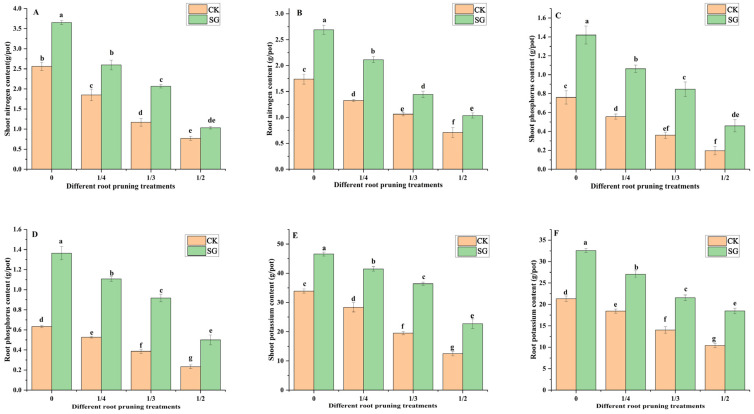
Effects of *Suillus grevillea* on the shoot and root nitrogen, phosphorus and potassium contents of seedlings. (**A**) The shoot nitrogen content; (**B**) The root nitrogen content; (**C**) The shoot phosphorus content; (**D**) The root phosphorus content; (**E**) The shoot potassium content; (**F**) The root potassium content; CK, without inoculation; SG, inoculation with *Suillus grevillea*; SG, inoculation with *Suillus grevillea*; the 0, 1/4, 1/3 and 1/2 represent the removal of zero, one-fourth, one-third, and one-half, respectively, of the seedling taproot length; different lowercase letters in the figure indicate that mean values are significantly different (*p* < 0.05) according to the LSD test, while the same letter denotes no significant difference between treatments; error bars represent the standard error (SE) of the mean (n = 5).

Nitrogen, phosphorus, and potassium are essential mineral nutrients for plant growth. As shown in Figure 4A–F, compared with those of the plants without root pruning, the shoot and root nitrogen, phosphorus and potassium contents of the inoculated *Quercus variabilis* Blume decreased with increasing degree of root pruning. These findings revealed that root pruning inhibits nutrient absorption by *Quercus variabilis* Blume seedlings. Under the 0, 1/4, 1/3 and 1/2 root pruning treatments, the shoot and root nitrogen, phosphorus, and potassium contents of inoculated seedlings were significantly greater than those without inoculation. In the 0, 1/4, 1/3, and 1/2 root pruning treatments, the shoot nitrogen content in the inoculated group of *Quercus variabilis* Blume seedlings increased significantly by 29.9%, 43.4%, 28.7%, and 25.5%, respectively, compared with that in the control groups.

Compared with that in noninoculated plants, the root nitrogen content significantly increased. Under root pruning treatments of 0, 1/4, 1/3, and 1/2, the shoot phosphorus content of seedlings inoculated with *Suillus grevillea* increased significantly compared with that of noninoculated plants. The root phosphorus content increased significantly compared to noninoculated plants. The shoot potassium content of inoculated *Quercus variabilis* Blume increased significantly by 27.3%, 31.6%, 46.4%, and 45% compared with that in noninoculated plants, whereas the root potassium content increased significantly by 34.5%, 31.8%, 34.9%, and 43.7% compared with that in noninoculated plants. *Suillus grevillea* promoted shoot and root absorption of nitrogen, phosphorus, and potassium in seedlings with different types of root pruning.

### 3.4. The Root Morphology of Seedlings

The root morphology reflects the root configuration and the ability of the plant to absorb and transport nutrients. As shown in Table 2, as the degree of root pruning increased, the root length, root projection area, root surface area, and root volume of noninoculated seedlings gradually decreased, whereas inoculation with *Suillus grevillea* alleviated the adverse effects of root pruning on the root morphology of *Quercus variabilis* Blume seedlings. When the degree of root pruning was 0 or 1/4, the root length, root projection area, root surface area, root average diameter, root density, root volume and root tips of the inoculated seedlings were significantly greater than those in the absence of root pruning (*p* < 0.05). Under the 1/3 or 1/2 root pruning treatment, compared with those of the noninoculated plants, the root length, root projection area, root surface area, and root volume of the inoculated *Quercus variabilis* Blume seedlings increased (*p* > 0.05). These results indicated that inoculation treatment significantly promoted root morphology development during mild root pruning but had a limited effect on improving the root morphology of *Quercus variabilis* Blume during severe root pruning. Thus, the degree of root pruning affects the effect of *Suillus grevillea* on the root morphology of seedlings.

### 3.5. Endogenous Hormones in the Roots of the Seedlings

**Figure 5:**
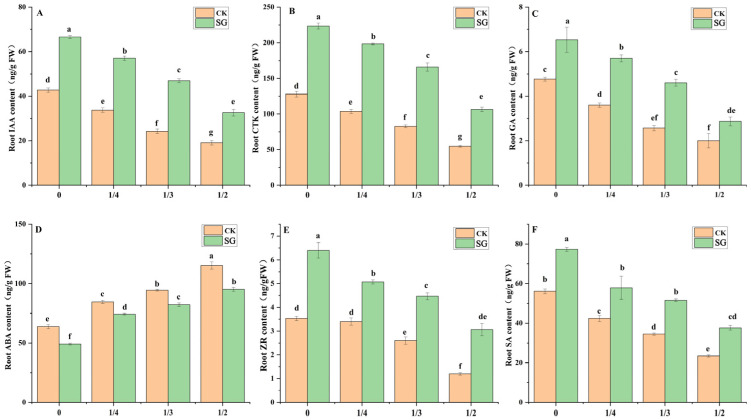
Effects of *Suillus grevillea* on the root endogenous hormone content of seedlings with different root pruning treatment. (**A**) The root endogenous IAA content of seedlings with different treatments; (**B**) The root CTK content of seedlings with different treatments; (**C**) The root endogenous GA content of seedlings with different root pruning treatments; (**D**) The root ABA content of seedlings with different treatments; (**E**) The root ZR content of seedlings with different root pruning treatments; (**F**) The root SA content of seedlings with different treatments; CK, without inoculation; SG, inoculation with *Suillus grevillea*; the values 0, 1/4, 1/3 and 1/2 represent the removal of zero, one-fourth, one-third, and one-half, respectively, of the seedling taproot length; different lowercase letters in the figure indicate that mean values are significantly different (*p* < 0.05) according to the LSD test, while the same letter denotes no significant difference between treatments; error bars represent the standard error (SE) of the mean (n = 5).

Endogenous hormones play a key role in regulating root physiological activities. Ectomycorrhizal fungi can induce the accumulation of root tip hormones in plants, thereby promoting the formation of fine roots. As the degree of root pruning increased, the root IAA, GA, CTK, ZR and SA contents of seedlings with and without inoculation significantly decreased (Figure 5); however, the root IAA, GA, CTK, ZR and SA contents of seedlings inoculated with *Suillus grevillea* significantly increased compared with those without inoculation. Under root pruning treatments of 0, 1/4, 1/3, and 1/2, the root IAA content of the inoculated *Quercus variabilis* Blume increased significantly (by 35.8%, 40.9%, 48.4%, and 41.5%, respectively) compared with that in the absence of inoculation, and the root CTK content of the inoculated seedlings increased significantly (by 42.8%, 47.8%, 50.2% and 48.5%, respectively). As the degree of root pruning increased, the root abscisic acid (ABA) contents of seedlings with and without inoculation significantly increased. Regardless of the degree of root pruning, the root ABA contents of the plants inoculated with *Suillus grevillea* were significantly lower than those of the plants without *Suillus grevillea*.

### 3.6. Correlations Between Root Morphology and Other Growth Indicators

**Figure 6:**
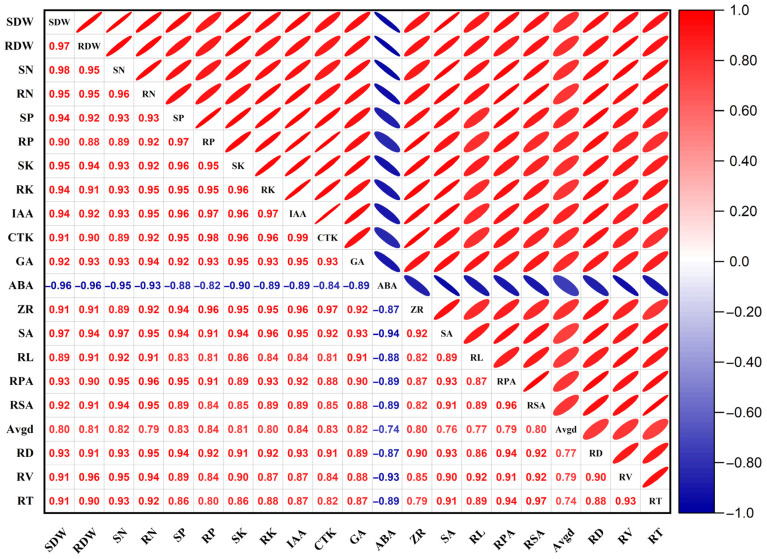
Correlations between the physiological parameters and growth of seedlings subjected to root pruning. SDW, Shoot dry weight; RDW, root dry weight; SN, shoot nitrogen content; RN, root nitrogen content; SP, shoot phosphorus content; RP, root phosphorus content; SK, shoot potassium content; RK, root potassium content; IAA, root auxin content; CTK, root cytokinin content; GA, root gibberellin content; ABA, root abscisic acid content; ZR, root ribosylzeatin content; SA, root salicylic acid content; RL, root length; RPA, root project area; RSA, root surface area; Avgd, average root diameter; RD, root density; RV, root volume; RT, root tips; the correlations among indicators, including aboveground biomass, root biomass, nutrient elements, endogenous hormones, and root morphology, were assessed using Pearson’s correlation coefficient.

As shown in Figure 6, the shoot dry weight (SDW) and root dry weight (RDW) of *Quercus variabilis* Blume seedlings were significantly correlated with the root length (RL), root projected area (RPA), root surface area (RSA), average root diameter (AvgD), root volume (RV) and shoot and root N, P, and K contents of the seedlings (*p* < 0.05). The SDW was significantly positively correlated with the shoot N content (r = 0.98), shoot P content (r = 0.94), shoot K content (r = 0.95), root N content (r = 0.95), root P content (r = 0.90), and root K content (r = 0.94) (*p* < 0.05). The RDW was significantly positively correlated with shoot N content (r = 0.95), shoot P content (r = 0.92), shoot K content (r = 0.94), root N content (r = 0.95), P content (r = 0.88), and K content (r = 0.91) (*p* < 0.05), RL (r = 0.91), RPA (r = 0.90), RSA (r = 0.91), Avgd (r = 0.81), RD (r = 0.91), RV (r = 0.96), and RT (r = 0.90) (*p* < 0.05), and the accumulation of biomass depended mainly on the supply of shoot and root nitrogen, phosphorus, and potassium.

There was a significant correlation between shoot and root biomass and endogenous hormone contents in the roots. The SDW was significantly correlated with IAA (r = 0.94), CTK (r = 0.91), GA (r = 0.92), ABA (r = −0.96), ZR (r = 0.91) and SA (r = 0.97), and the RDW was significantly correlated with IAA (r = 0.92), CTK (r = 0.90), GA (r = 0.93), ABA (r = −0.96), ZR (r = 0.91) and SA (r = 0.94). RV was significantly positively correlated with the RL (r = 0.92), RPA (r = 0.91), RSA (r = 0.92), AvgD (r = 0.79), RD (r = 0.90) and RD (r = 0.93) of the seedlings, indicating that the root morphological parameters were closely correlated and regulated by endogenous root hormones.

The detailed values for Cronbach’s Alpha (CA), composite reliability (CR), average variance extracted (AVE), and factor loadings in the PLS-SEM model are presented in Table 3. These results indicate that the constructed PLS-SEM model demonstrates adequate reliability and validity to satisfy the analytical requirements of this study.

**Figure 7:**
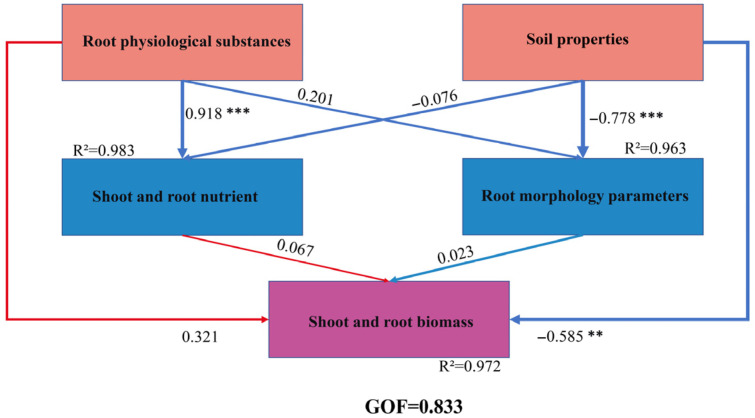
Path coefficients and significance of various correlated factors affecting the growth of root pruning seedlings. R^2^ represents the explanatory power of the variable, the number represents the path coefficient, GOF represents the goodness of fit, ** represents significant at the 0.01 level, and *** represents significant at the 0.001 level.

To analyze the indirect and direct pathways influencing the growth of root-pruned *Quercus variabilis* Blume seedlings, this study developed a partial least squares structural equation model (PLS-SEM) to elucidate the regulatory pathways governing their growth (Figure 7). The goodness-of-fit (GOF) value was 0.833, which indicates an adequate overall model fit. The results indicated that root physiological substances exerted a highly significant positive direct effect on both shoot and root nutrient contents (r = 0.918 ***, R^2^ = 0.983), whereas their direct effect on root morphological parameters was non-significant (r = 0.201). Concurrently, root physiological substances had a moderate positive direct effect on both shoot and root biomass (r = 0.321). Soil properties exerted a highly significant negative direct effect on root morphological parameters (r = −0.778 ***, R^2^ = 0.963), whereas their direct effects on aboveground and root nutrients were non-significant (r = −0.076). Soil properties also exerted a significant negative direct effect on both aboveground and root biomass (r = −0.585 **). The direct effects of aboveground and root nutrients (r = 0.067) and of root morphological parameters (r = 0.023) on biomass were both non-significant.

### 3.7. Soil Properties

As shown in Table 4, ECMF are important soil microorganisms that play crucial roles in soil nutrient cycling and organic matter degradation. under the 0, 1/4, and 1/2 root pruning treatments, the soil pH value in the M chamber was the lowest, and that in the R chamber was the highest. As the degree of root pruning increased, the soil pH and soil electrical conductivity decreased. Regardless of whether the seedlings were inoculated or noninoculated, the soil electrical conductivity in the M chamber was the highest, and that in the R chamber was the lowest.

The soil available phosphorus and potassium contents significantly increased as the root pruning degrees increased (*p* < 0.05). When the degree of root pruning was 0, 1/4, 1/3 and 1/2, the soil available phosphorus and potassium contents and soil alkaline nitrogen content in the M chamber were the highest, and those in the R chamber were the lowest. As the degree of root pruning increased, the soil alkaline nitrogen content, total nitrogen content and soil total carbon content gradually decreased. The total nitrogen and carbon contents of the soil in the three chambers were the highest in the M chamber, followed by the hyphal chamber, and finally the root chamber.

**Table 4:** Soil properties of three departments.

Inoculation Treatment	Different Root Pruning Treatments	pH	Conductivity (µS/cm)	Available Phosphorus Content (mg/kg)	Available Nitrogen Content (mg/kg)	Available Potassium Content (mg/kg)	Total Nitrogen Content (%)	Total Carbon Content (%)
R	0	7.90 ± 0.03 a	484.33 ± 4.63 cd	18.91 ± 0.33 g	535.33 ± 4.06 g	13.76 ± 0.9 f	0.37 ± 0.01 g	2.46 ± 0.06 f
M	0	6.87 ± 0.12 f	794.67 ± 30.9 a	32.43 ± 0.67 e	916.67 ± 23.97 a	31.52 ± 0.44 c	0.84 ± 0.01 a	4.68 ± 0.10 a
H	0	7.35 ± 0.06 c	629.67 ± 51.85 b	26.11 ± 1.06 f	840 ± 8.14 b	28.05 ± 0.89 d	0.64 ± 0.05 d	3.68 ± 0.05 c
R	1/4	7.58 ± 0.03 b	354 ± 9.85 e	27.69 ± 1.23 f	623.67 ± 6.94 f	22.74 ± 0.88 e	0.32 ± 0.01 gh	1.81 ± 0.08 g
M	1/4	6.59 ± 0.07 g	641.33 ± 40.4 b	47.76 ± 1.35 a	836.67 ± 11.14 b	34.4 ± 0.95 b	0.78 ± 0.01 b	4.03 ± 0.05 b
H	1/4	7.17 ± 0.04 de	551 ± 19.52 c	36.41 ± 1.38 d	750.33 ± 0.67 c	31.73 ± 0.39 c	0.64 ± 0.01 d	3.36 ± 0.10 d
R	1/3	7.24 ± 0.05 cd	257.67 ± 2.03 f	34.96 ± 1.17 de	712 ± 7.37 d	26.45 ± 0.62 d	0.28 ± 0.01 hi	1.47 ± 0.04 h
M	1/3	6.44 ± 0.06 g	459 ± 17.78 d	47.35 ± 1.06 a	824.67 ± 6.64 b	37.52 ± 0.56 a	0.72 ± 0.02 c	3.9 ± 0.03 b
H	1/3	7.02 ± 0.02 ef	387.67 ± 14.45 e	38.26 ± 1.21 cd	766.67 ± 12.72 c	33.41 ± 0.53 bc	0.63 ± 0.01 d	3.14 ± 0.04 e
R	1/2	7.02 ± 0.02 ef	163.33 ± 19.53 g	40.29 ± 0.58 c	668.33 ± 8.67 e	32.33 ± 0.58 c	0.24 ± 0.01 i	1.29 ± 0.12 h
M	1/2	6.23 ± 0.04 h	340 ± 10.02 e	44.32 ± 0.64 b	761 ± 6.24 c	37.44 ± 0.66 a	0.56 ± 0.01 e	2.99 ± 0.02 e
H	1/2	6.90 ± 0.04 f	233.33 ± 12.03 f	35.89 ± 0.63 d	701 ± 5.69 d	34.54 ± 0.68 b	0.46 ± 0.02 f	2.52 ± 0.03 f

Note: R, root chamber; M, mycorrhizal chamber; H, hyphal chamber; the values 0, 1/4, 1/3 and 1/2 represent the removal of zero, one-fourth, one-third, and one-half, respectively, of the seedling taproot length; the data presented in the table are expressed as mean values ± standard error; different lowercase letters in the figure indicate that mean values are significantly different (*p* < 0.05) according to the LSD test, while the same letter denotes no significant difference between treatments.

## 4. Discussion

### 4.1. Effects of Suillus grevillea on the Physiological Growth of Seedlings

The shoot and root dry weights directly reflect the quality of afforested plants [41]. In this study, root pruning reduced the shoot and root dry weights of *Quercus variabilis* Blume seedlings; this may have occurred because root pruning reduces the ability of the roots to absorb nutrients and water [8]. Root damage significantly delays the leaf unfolding initiation stage and full leaf unfolding stage [7]. Vaccination with *Suillus grevillea* can alleviate these adverse effects. In this study, *Suillus grevillea* inoculation improved the shoot and root growth of *Quercus variabilis* Blume seedlings. Moreover, *Suillus grevillea* inoculation improved nitrogen, phosphorus, and potassium absorption in the seedlings in the different root pruning treatments (Figure 3). *Suillus grevillea* expands the absorption of nutrients by the root system through hyphae [42]. Mycelia are finer than roots and can reach soil pores that are difficult for roots to reach, absorbing more water and nutrients, especially essential elements such as phosphorus and nitrogen, to compensate for the decrease in absorption capacity caused by root pruning [43].

Root system architecture is an important developmental and agronomic trait, with implications for overall plant architecture, growth rate and yield, abiotic stress resistance, nutrient uptake, and developmental plasticity in response to environmental changes [16]. Root architecture is modulated by intrinsic, hormone-mediated pathways, intersecting with pathways that perceive and respond to external, environmental signals [16]. The root morphological structure determines the ability of plants to absorb and transport nutrients [44]. In this study, the effect of inoculation with *Suillus grevillea* on improving the root morphology of *Quercus variabilis* is closely associated with root pruning intensity. Under 0 and 1/4 root pruning treatments, this symbiotic interaction exhibits a prominent promoting effect on root morphology, whereas as pruning intensity increases to 1/3 and 1/2 root pruning treatments, the promotional effect diminishes significantly (Table 2). This is because mild root pruning causes minimal damage to the root system, moderately stimulates lateral root formation in seedlings, and enhances the root system’s capacity to absorb soil nutrients and water—thereby improving seedling survival rate [4]. In contrast, severe root pruning results in a substantial reduction in root biomass and absorptive surface area, rendering the plant unable to efficiently uptake water and mineral nutrients. Concurrently, the plant prioritizes allocating photosynthates to root wound repair, which disrupts the root-shoot growth balance and redirects limited resources toward healing rather than synergistically establishing optimized root morphology with *Suillus grevillea* [45]. The root length, root projection area, root surface area, root average diameter, root density, root volume and root tips of oak trees inoculated with *Suillus grevillea* were greater than those of the uninoculated group regardless of the degree of root pruning, which is consistent with the results of [45], possibly because of the physiological substances produced by ectomycorrhizal fungi after root pruning [46]. Root pruning resulted in the production of more main root branches and greater surface area values for fine and coarse roots [20]. This may be because *Quercus variabilis* Blume seedlings promote lateral root development and improve root structure by activating the secretion of endogenous hormones (such as IAA, CTK, and ABA) and regulating the expression of 20 key genes after root pruning [3]. These findings can be explained by the correlations between root morphology parameters and root endogenous hormones in this study. The gradient of IAA in plant root tips determines the orientation of roots and affects their vertical growth and development, and the metabolism-related gene CKX2 regulates root CTK to promote root cell proliferation and affect lateral root growth, and the antagonistic effects of these genes affect root structure [47]. Several studies have demonstrated that root symbiotic fungi can modify the root architecture of host plants, thereby increasing plant growth performance [48,49].

In this study, different degrees of root pruning affected the endogenous hormone content in the root system of the seedlings, and *Suillus grevillea* regulated changes in root endogenous hormones and improved seedling growth (Figure 4). Decreases in IAA, CTK, ZR, SA and GA concentrations were considered ecological strategies used by *Quercus variabilis* Blume seedlings to resist root pruning. Following root pruning, IAA accumulates in the adventitious root stem cell niche, whereas CTK accumulates in the root cap and vasculature regions, and the spatial distributions of these hormones are mutually exclusive [50]. Inoculation with *Suillus grevillea* increased the root IAA, GA and CTK contents of seedlings in response to root pruning treatments (Figure 4), which is consistent with previous research findings that ECMF promotes host plants in adapting to unfavorable environments by regulating hormones [29,51].

In this study, compared with that in inoculated seedlings, the ABA concentration in noninoculated seedlings was significantly greater under different degrees of root pruning. This finding indicates that the increase and decrease in the different hormones did not inhibit growth as expected, possibly because of the mitigating effect of mycorrhizal inoculation on the physiological response of the plant to stress [41].

### 4.2. Effects of Suillus grevillea on the Rhizosphere Soil Properties of Seedlings

Soil pH is among the key factors affecting soil nutrient availability and microbial community structure [52,53]. In this study, *Suillus grevillea* reduced the soil pH, which is consistent with previous results [54,55]. ECMF secretes acidic substances from seedling roots after pruning, activating insoluble mineral substances in the soil [56]. In addition, ectomycorrhizal fungi secrete organic acids (such as oxalic acid, citric acid, and acetic acid) and protons (H^+^) from their outer hyphae. Acidic substances can diffuse several centimeters beyond the rhizosphere and increase soil acidity [57]. In addition to organic acids, ectomycorrhizal fungi further acidify the soil microenvironment through the release of protons (H+) on the plasma membrane [58].

In this study, the soil available phosphorus content, available nitrogen content, and available potassium content in the M chamber were greater than those in the R and H chambers. ECMF can secrete acid phosphatases, which can hydrolyze insoluble organic phosphides and convert them into available inorganic phosphorus, thereby accelerating soil nutrient cycling [22]. In addition, mycorrhizal hyphae also secrete proteases and aminopeptidases, which can decompose proteins in soil organic matter and gradually degrade them into free amino acids, which are converted into ammonium or nitrate nitrogen that plants can absorb through ammonification or nitrification, thereby accelerating soil nitrogen cycling and increasing the content of alkaline hydrolyzed nitrogen [59]. In this study, the total carbon and total nitrogen contents in the rhizosphere soil of oak seedlings inoculated with *Suillus grevillea* were greater than those in the rhizosphere soil without *Suillus grevillea*, and these results are consistent with those of [60]. Ectomycorrhizal fungi transport host plant photosynthetic products (such as glucose and sucrose) to the roots and mycelia, where they produce cellulases and ligninases, promote microbial decomposition of soil organic matter and root exudates, and release more carbon into the soil [61].

*Suillus grevillea* demonstrates both similarities and differences in its regulation of rhizosphere soil properties across various host tree species. *Suillus grevillea* typically modulates the soil environment through various physiological and metabolic pathways, including secretion of organic acids to lower soil pH, release of phosphatase to activate soil-bound phosphorus (particularly organic phosphorus), and enhancement of nutrient availability. Host-specific differences in the intensity and functional focus of *Suillus grevillea’s* regulatory effects vary across tree species. Inoculation with *Suillus grevillea* significantly increases total nitrogen (TN) and total phosphorus (TP) content in the leaves of *Quercus nuttallii*, with particularly notable improvements in phosphorus availability, TP content in inoculated leaves reaching 2.72 g/kg, significantly higher than the uninoculated control (2.08 g/kg). This outcome primarily reflects the fungus’s facilitation of the release of readily available nitrogen and phosphorus through the aforementioned mechanisms, thereby enhancing host absorption [62]. Following inoculation with *Suillus grevillea*, seedlings of *Pinus thunbergii* and *Quercus acutissima* became more dependent on mycorrhizal symbiotic structures for indirect regulation of soil properties. The strain secreted hormones, such as indole-3-acetic acid (IAA), to promote host root development (e.g., lateral root proliferation, increased root density), thereby enhancing soil nutrient cycling efficiency. Concurrently, it optimized the stability of the soil microenvironment through its mycelial network, thereby indirectly enhancing the capacity for nutrient supply [63]. In the case of *Pinus tabulaeformis*, *Suillus grevillea’s* regulatory function is focused on heavy metal remediation. Through biomineralization, it converts toxic Pb^2+^ in the soil into thermodynamically stable chloropyrophosphate (Pb_5_(PO_4_)_3_Cl), significantly reducing Pb bioavailability and migration risks, while simultaneously improving the soil microenvironment to support host growth [64]. In the case of *Pinus massoniana*, *Suillus grevillea* exhibits potent organic phosphorus mineralization capacity while enhancing the activities of soil acid phosphatase, alkaline phosphatase, and phytase [].

Different ectomycorrhizal fungi exert varying regulatory effects on the rhizosphere soil properties of *Quercus variabilis* Blume, with differences in regulatory intensity and functional emphasis depending on the fungal type. Ectomycorrhizal fungi (*Russula*, *Inocybe*, *Cenococcum*, *Scleroderma*) influence soil physicochemical properties and nutrient availability through their regulatory effects []. Ectomycorrhizal fungi (*Pisolithus sp*, *Cenococcum geophilum*, *Laccaria*) promote the survival, growth, and nutrient uptake of *Quercus variabilis* Blume seedlings through the regulation of biomass allocation processes [65].

Although the positive effects of *Suillus grevillea* on the growth of root-pruned *Quercus variabilis* Blume seedlings and the physicochemical properties of the rhizosphere soil have been clarified in this study. However, examining the physiological effects of a single ectomycorrhizal fungus on *Quercus variabilis* growth without comparative analysis against multiple ectomycorrhizal fungi results in a somewhat one-sided analysis. Furthermore, the influence of ectomycorrhizal fungi on *Quercus variabilis* remains confined to the physiological level, failing to elucidate the growth regulation mechanisms of ectomycorrhizal fungi on root-pruned seedlings at the biomolecular level. The number of soil variables analyzed is relatively low to support the claim that ectomycorrhizal fungi affect rhizosphere soil properties.

## 5. Conclusions

In this study, the effects of *Suillus grevillea* inoculation on the growth, nutrient absorption, root endogenous hormone level, root morphology and rhizosphere soil properties of *Quercus variabilis* Blume seedlings were analyzed. Inoculation with *Suillus grevillea* can alleviate the adverse effects of root pruning on the shoot and root dry weight of plants. Under the 0, 1/4, 1/3 and 1/2 root pruning treatments, *Suillus grevillea* inoculation significantly promoted the absorption of nitrogen, phosphorus and potassium by the shoot and root of the plants. Under the 0 and 1/4 root pruning treatments, *Suillus grevillea* improved the root morphology parameters (root length, root projection area, root surface area, average root diameter, root density, root volume and root tips) of *Quercus variabilis* Blume seedlings. *Suillus grevillea* inoculation regulated the root IAA, CTK, GA, ABA, ZR and SA contents of the seedlings after root pruning and reduced the adverse effects of root pruning on root physiology. *Suillus grevillea* can reduce soil acidity and activate insoluble minerals. The increased soil available phosphorus, nitrogen, potassium, and total nitrogen contents improved the rhizosphere soil environment of the root-pruned seedlings. Future research should prioritize investigating the molecular mechanisms by which ectomycorrhizal fungi (ECMF) regulate the growth of root-pruned seedlings, thus providing a theoretical framework to accelerate the application of mycorrhizal technology in forest cultivation.

## Figures and Tables

**Table 1:** Mycorrhizal colonization and mycelial density of *Quercus variabilis* Blume seedlings under different root pruning treatments.

Inoculation Treatment	Different Root Pruning Treatments	Mycorrhizal Colonization (%)	Mycelial Density (g/cm)
CK	0	0.00 ± 0.00 e	0.00 ± 0.00 e
SG	0	86.6 ± 9.07 a	6.83 ± 0.61 a
CK	1/4	0.00 ± 0.00 e	0.00 ± 0.00 e
SG	1/4	63.33 ± 3.33 b	3.58 ± 0.32 b
CK	1/3	0.00 ± 0.00 e	0.00 ± 0.04 e
SG	1/3	43.4 ± 1.95 c	2.95 ± 0.05 c
CK	1/2	0.00 ± 0.00 e	0.00 ± 0.00 e
SG	1/2	29.9 ± 4.51 d	1.72 ± 0.18 d

Note: CK, without inoculation; SG, inoculation with Suillus grevillea; the values 0, 1/4, 1/3 and 1/2 represent the removal of zero, one-fourth, one-third, and one-half, respectively, of the seedling taproot length; the data presented in the table are expressed as mean values ± standard error; different lowercase letters in the table indicate that mean values are significantly different (*p* < 0.05) according to the LSD test, while the same letter denotes no significant difference between treatments.

**Table 2:** Effects of *Suillus grevilleae* on the root morphology of seedlings with different root pruning treatments.

Inoculation Treatment	Different Root Pruning Treatments	RL (cm)	RPA (cm^2^)	RSA (cm^2^)	ARD (mm)	RD (cm/m^3^)	RV (cm^3^)	RT
CK	0	315.79 ± 3.41 bc	50.64 ± 3.22 c	231.95 ± 4.86 c	1.74 ± 0.26 bcd	45.81 ± 1.89 c	5.76 ± 0.34 b	1545 ± 151.31 b
SG	0	397.38 ± 31.64 a	92.05 ± 1.12 a	362.65 ± 19.30 a	2.58 ± 0.09 a	82.69 ± 5.55 a	8.19 ± 0.40 a	2144.67 ± 298.14 a
CK	1/4	277.10 ± 1.86 cd	31.46 ± 0.53 d	145.49 ± 9.56 d	1.57 ± 0.13 cd	35.78 ± 1.44 cd	4.63 ± 0.14 c	824.67 ± 36.04 c
SG	1/4	342.06 ± 15.81 b	60.97 ± 3.47 b	277.69 ± 7.95 b	2.29 ± 0.35 ab	66.58 ± 8.08 b	6.23 ± 0.53 b	1684.33 ± 107.91 b
CK	1/3	247.44 ± 13.81 d	20.51 ± 0.55 e	108.99 ± 8.80 ef	1.34 ± 0.27 cd	28.43 ± 4.42 de	3.55 ± 0.23 d	451.67 ± 15.98 cd
SG	1/3	272.46 ± 15.41 cd	36.65 ± 4.57 d	132.29 ± 18.84 de	1.92 ± 0.04 bc	43.08 ± 0.73 c	4.36 ± 0.15 cd	781.67 ± 26.61 c
CK	1/2	172.17 ± 10.27 e	19.34 ± 0.26 e	86.58 ± 1.46 f	1.17 ± 0.04 d	17.09 ± 2.23 e	1.50 ± 0.15 e	301.33 ± 34.65 d
SG	1/2	176.28 ± 6.97 e	23.40 ± 1.34 e	91.59 ± 2.24 f	1.23 ± 0.03 d	20.79 ± 0.31 e	1.95 ± 0.13 e	375.33 ± 11.79 d

Note: CK, without inoculation; SG, inoculation with *Suillus grevillea*; the values 0, 1/4, 1/3 and 1/2 represent the removal of zero, one-fourth, one-third, and one-half, respectively, of the seedling taproot length; RL, root length; RPA, root project area; RSA, root surface area; ARD, average root diameter; RD, root density; RV, root volume; RT, root tip number; the data presented in the table are expressed as mean values ± standard error; different lowercase letters in the figure indicate that mean values are significantly different (*p* < 0.05) according to the LSD test, while the same letter denotes no significant difference between treatments.

**Table 3:** Reliability and validity assessment of latent variables and their corresponding observed variables in partial least squares structural equation modeling (PLS-SEM).

Latent Variable	Observed Variable	Loading	CA	CR	AVE
Root physiological substances	IAA	0.987	0.983	0.968	0.989
	CTK	0.971			
	GA	0.967			
	ABA	0.936			
	ZR	0.971			
	SA	0.975			
Soil properties	Conductivity	0.949	0.780	0.693	0.580
	TC	0.750			
	TN	0.695			
Shoot and root nutrient	Shoot Nitrogen	0.965	0.989	0.974	0.949
	Root Nitrogen	0.971			
	Shoot Phosphorus	0.980			
	Root Phosphorus	0.970			
	Shoot Potassium	0.977			
	Root Potassium	0.982			
Root morphological parameters	RL	0.939	0.986	0.968	0.894
	RPA	0.969			
	RSA	0.976			
	ARD	0.851			
	RD	0.951			
	RV	0.964			
	RT	0.962			

Note: CA, Cronbach’s Alpha; CR, Composite Reliability; AVE, Average Variance Extracted; IAA, Indole-3-acetic acid; CTK, Cytokinin; GA, Gibberellin; ABA, Abscisic acid; ZR, Zeatin riboside; SA, Salicylic acid; Conductivity, Soil electrical conductivity; TC, Total carbon; TN, Total nitrogen; RL, Root length; RPA, Root Projection Area; RSA, Root surface area; ARD, Average root diameter; RD, Root diameter; RV, Root volume; RT, Root tips; CA value > 0.7, CR value > 0.7 and AVE value > 0.5 indicate acceptable reliability and validity of the latent constructs.

## Data Availability

The data that support the findings of this study are available on request from the corresponding author, Liu Yang (yangliu@henau.com.cn), upon reasonable request due to (specify the reason for the restriction).
